# Rectal aberrant crypt foci (ACF) as a predictor of benign and malignant neoplastic lesions in the large intestine

**DOI:** 10.1186/s12885-020-6590-4

**Published:** 2020-02-19

**Authors:** Marek Kowalczyk, Marcin Orłowski, Łukasz Klepacki, Krzysztof Zinkiewicz, Waldemar Kurpiewski, Dorota Kaczerska, Wiesław Pesta, Ewa Zieliński, Piotr Siermontowski

**Affiliations:** 1 Department of Oncologic and General Surgery, University Hospital in Olsztyn, Olsztyn, Poland; 2 Department of Laboratory Medicine, University Hospital in Olsztyn, Olsztyn, Poland; 3 Centre for Diagnosis and Treatment of Gastrointestinal Diseases, Gdańsk, Poland; 4 Department of Anatomy, University Hospital in Olsztyn, Olsztyn, Poland; 5 Oncological and General Surgery Clinic, University Hospital in Olsztyn, Olsztyn, Poland; 6 52nd Department of General, Gastroenterologic and Gastrointestinal Oncologic Surgery, Medical University of Lublin, University Hospital No.1, Lublin, Poland; 7 Department of Oncologic and General Surgery, University Hospital in Olsztyn, Olsztyn, Poland; 8 WSB University of Gdańsk, Gdańsk, Poland; 9 Department of Oncologic and General Surgery, University Hospital in Olsztyn, Olsztyn, Poland; 10 Department of Emergency Medicine and Disaster Collegium Medicum in Bydgoszcz, Nicolaus Copernicus University in Toruń, Toruń, Poland; 11 Department of Underwater Works Technology, Polish Naval Academy, Gdynia, Poland; 12 Department of Maritime & Hyperbaric Medicine Department, Military Institute of Medicine Gdynia, Warsaw, Poland

**Keywords:** Large intestine, Benign and malignant neoplastic lesions, Rectal ACF

## Abstract

**Background:**

The importance of ACF is not fully explained, however, their number may be a good predictor of synchronous and metachronic adenoma or other polyps whose removal reduces the risk of CRC. Due to the epidemiological and genetic association of ACF with pre-cancer lesions, they may be a potential CRC biomarker. The aim of our study was to show that the number and type of rectal ACF may be a good predictive factor for the presence of polyps located proximally from the splenic flexure and that the type and number of ACF can correlate with the number and specific types of polyps in the large intestine.

**Methods:**

The study included 131 patients who underwent colonoscopy combined with rectal mucosa staining with 0.25% methylene blue. The number of rectal ACF was determined and bioptats were sampled for histopathological examination to assess the type of ACF. Endoscopic ACF assessment criteria given by L. Roncucci were used. The obtained material was subjected to statistical analysis using probability distribution, U-test, t-student test, and chi ^2^ as well as the Statistica 7.1 software package.

**Results:**

The study population was divided into three subgroups according to the number of ACF observed, i.e. ACF < 5, 5–10 and > 10. ACF < 5 were found in 35 patients (29.41%), 5–10 ACF in 70 (58.82%) and ACF > 10 in 14 individuals (11.76%).

The study revealed the presence of normal ACF (*p* = 0.49), hyperplastic ACF (*p* = 0.34), dysplastic ACF (*p* = 0.11), and mixed ACF (*p* = 0.06). A single type of ACF was most commonly observed (*n* = 88, *p* = 0.74). In the researched group a larger number of ACF is concurrent with adenomas and hyperplastic polyps. The number of ACF clearly correlates with the dysplasia advancement in the adenoma and the number of polyps found.

**Conclusions:**

Rectal ACF are a useful marker for the presence of cancerous lesions in the proximal and distal sections of the large intestine.

## Background

The process of development of colorectal cancer is preceded by an occurrence of indirect lesions of which only a small part are subject to malignant transformation. While environmental factors may favour the development of colorectal cancer, pre-cancerous conditions constitute a real threat of its occurrence. Modern medicine aims at the earliest possible detection and treatment of pre-cancerous conditions manifested in the form of polyps of the large intestine. The main clinical significance with regard to their incidence is attributable to adenoma and serrated polyps. Moreover, there have been reports on the risk of neoplastic transformation of hyperplastic polyps, however, it is considered a low risk [1–3].

According to Vogelstein’s theory, carcinogenesis of the large intestine involves a sequence of changes from a healthy mucous membrane, through excessive proliferation and aberrant crypt foci (ACF), to the formation of adenoma and its malignant transformation. ACF are the earliest developing precursors of epithelial neoplasms of the large intestine. However, numerous studies have shown that mutations in the three “classic” genes of the Fearon-Vogelstein model, i.e. APC, KRAS and P53, are observed in slightly over 10% of cases of colorectal cancer [4]. In the remaining cases changes need to occur in other genes, cell metabolism and physiology thus leading to a fully malignant phenotype [5].

Numerous studies have shown that individuals with adenomas and colorectal cancer (CRC) have a higher percentage of ACF as compared to patients without a similar pathology in the large intestine. It is important that in some studies ACF were found in the direct vicinity of adenomas as were adenomas found at the periphery of CRC [6, 7]. An analysis of various studies shows that dysplastic ACF most commonly accompany adenomas and CRC [6].

Due to the epidemiological and genetic relationship between ACF and CRC, they are a potential CRC biomarker.

Detection of early lesions and their removal during colonoscopy (ACF, large intestine polyps) is a preventive method serving the improvement of treatment results, as in the case of cancers detected in the asymptomatic period (e.g. carcinoma in situ) the prognosis is much better. It should also be remembered that clinical symptoms are the most common symptoms of advanced cancers following a long-lasting asymptomatic course, and in these cases the prognosis is definitely worse. The significance of the number of ACF is not fully known, however it may be a good predictor of synchronous and metachronic adenoma or other polyps whose removal reduces the risk of CRC.

The aim of our study was to demonstrate that the number and type of rectal ACF can be a good predictor of the presence of polyps proximal to the splenic flexure and that the type and number of ACF may correlate with the number and specific types of polyps in the large intestine.

## Methods

The study encompassed 131 patients who underwent colonoscopy combined with rectal mucosa staining with 0.25% methylene blue. The study group consisted of 73 women and 58 men. The average age in the study group with regard to women was 67 years, whereas to men 52 years. The study was approved by the Bioethical Committee at the Faculty of Medical Sciences of the University of Warmia and Mazuria in Olsztyn – Resolution No. 11/2010 as of April 29, 2010.

The study was carried out using the Olympus-CF Q-165 L video colonoscope, Olympus FB-240 U biopsy forceps and the Olympus polyethylene spray catheter. After performing a routine colonoscopy and assessing all identified pathologies in the examined large intestine, the mucosa of the rectum in the section between the pecten analis and the central Huston valve was stained with 0.25% methylene blue solution and assessed with regard to the presence of ACF.

The number of rectal ACF was determined and bioptates were taken for the purpose of carrying out a histopathological examination to determine the ACF type. The endoscopic ACF assessment criteria defined by Roncucci were applied [8].

In the statistical analysis, the results were presented as numerical data, mean values, standard deviation, and probability distribution. In order to determine the statistical significance of results the U-test, t-student test, chi^2^ were used. The software used in the analysis was Statistica 7.1 and Excel 2010.

## Results

The study population was divided into three subgroups according to the number of ACF observed, i.e. ACF < 5, 5–10 and > 10. ACF < 5 were found in 35 patients (29.41%), 5–10 ACF in 70 (58.82%) and ACF > 10 in 14 individuals (11.76%).

In each subject the researchers identified one or several types of ACF. The most common case involved the presence of a single ACF type (*n* = 88). Various types of ACF were observed in a single polyp type.

In the examined group, normal ACF occurs with relative frequency f/*n* = 0,49, hyperplastic ACF f/n = 0,33, dysplastic ACF f/n = 0,1, mixed ACF f/n = 0,08.

### Occurrence of polyp types, adenoma size and dysplasia in the adenoma depending on the number of ACFs in the rectum (Table 1, Fig. 1)

Probability of single polyp occurence is bigger for people with smaller number of ACF. Decrease of polyp amount in that group ACF (ACF < 5) is exponential, which may indicate the initial periods of the neoplasmic process (early stage) Maximum relative frequency for that group ACF =0,48.

**Table 1:** The table shows the number of polyps, type of polyps, size of the adenoma, dysplasia in the adenoma, in the study group depending on the number of ACF in the rectum

	ACF < 5				ACF 5–10				ACF > 10			
Number of polyps	0	1	2	> 3	0	1	2	> 3	0	1	2	> 3
Number of people (relative frequention)	17 (0.48)	12 (0.34)	5 (0.14)	1 (0.02)	12 (0.17)	21 (0.3)	23 (0.32)	14 (0.2)	0	3 (0.21)	4 (0.21)	7 (0.5)
Type of polyp	No polyp	Adenoma	Hyperplastic	Serrated	No polyp	Adenoma	Hyperplastic	Serrated	No polyp	Adenoma	Hyperplastic	Serrated
Number (relative frequency)	22 (0.64)	3 (0.08)	8 (0.23)	1 (0.03)	7 (0.07)	33 (0.35)	44 (0.47)	9 (0.09)	0	12 (0.48)	10 (0.4)	3 (0.12)
Adenoma size	< 10 mm	10-20 mm		> 20 mm	< 10 mm	10-20 mm		> 20 mm	< 10 mm	10-20 mm		> 20 mm
Number	2	1		0	13	18		2	0	4		8
Degree of dysplasia	low		high		low		high		low		high	
Number	3		0		28		5		4		8	
Sex	man		woman		man		woman		man		woman	
Number	16		19		32		38		4		10

**Fig. 1:**
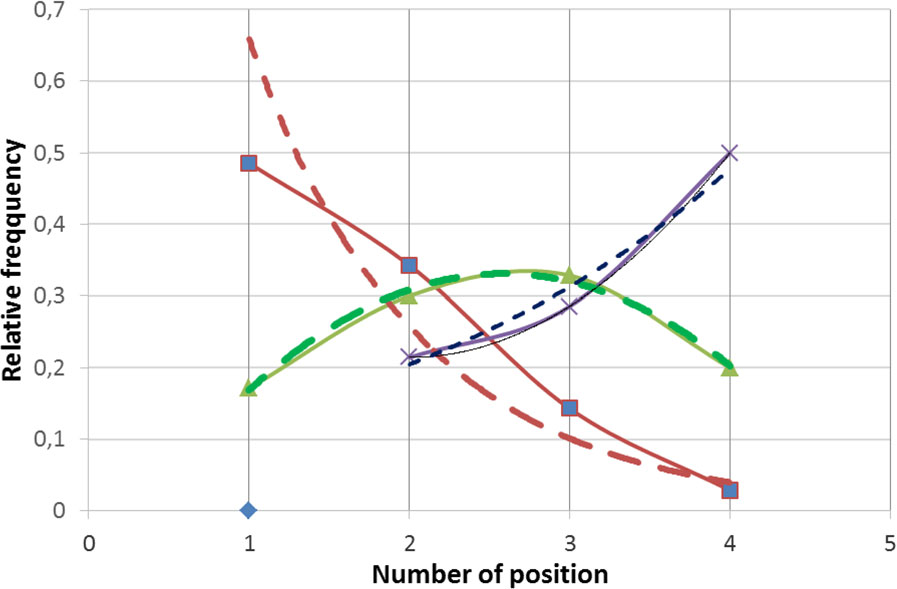
Probability of polyps occurence in a large intestine depending on the type of ACF in the rectum. 1- no polyp, 2- one polyp, 3-two polyps, 4- three and more polyps Red line- ACF < 5, Green line 5 < ACF < 10, Blue line-ACF > 10

On the basis of the curve for ACF < 5 one can state the type of the relative frequency distribution of an exponential curve in the form of the equation y = 1682e to the power − 0,938x *y* = 1.682*e*^−0, 938*x*^ z with a high indicator *R*^2^ = 0, 9166.For the group 5 < ACF < 10 the probability of polyp occurrence in a bigger amount increases and then it decreases according to the normal distribution. Maximum relative frequency for that group is = 0,328.

**Fig. 2:**
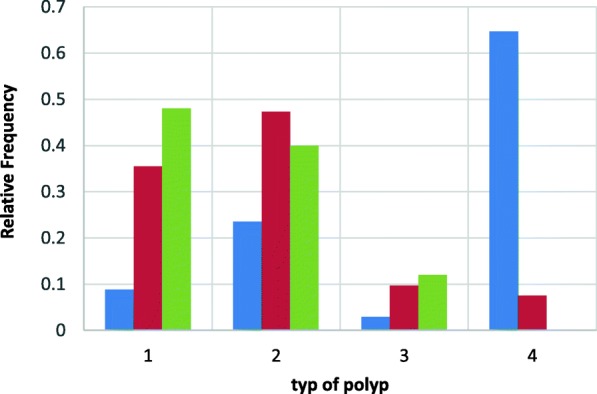
The probability of developing polips. 1-adenoma, 2- hyperplastic polyp, 3-serated polyp, 4- no polypsBlue- ACF < 5, Red-5 < ACF < 10, Green-ACF > 10

For the group ACF > 10 there is a bigger probability of occurrence of the bigger amount of polyps than in other groups and that increase has exponential characters in the shape of the equation *y* = 0, 0305*e*^1, 0397*x*^ with the determination indicator *R*^2^ = 0, 9643 Maximum relative frequency for that group is 0,5 and the curve character showing that group ACF can prove the progress and bigger dynamics of neoplasmic process. This suggests paying special attention to such patients in terms of the frequency of performing control colonoscopies.High determination rates in the study groups testify a good fit of the theoretical model to the experimental data (Fig. 2).

The probability of developing adenoma is the lowest for the group with a lower ACF number (ACF < 5) and the probability increases as the number of ACF increases. For the group ACF < 5 and ACF > 10 this difference is statistically significant with a significance level of *p* < 0,05With the increase in the number of ACF, the type of co-occurring polyps in the large intestine and their probability distribution change.

**Fig. 3:**
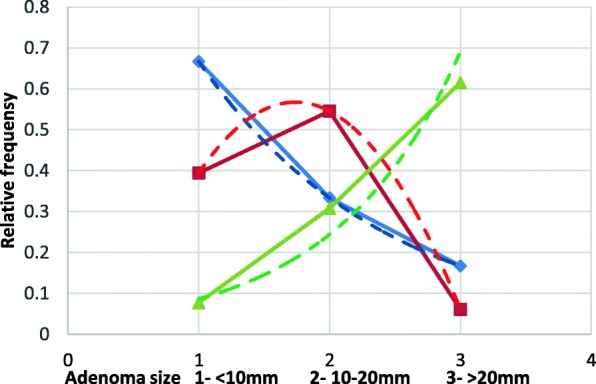
Probability of adenoma size depending on the number of rectal ACF (up to 10 mm). Blue line ACF < 5, Red line-5 < ACF < 10, green line ACF > 10

For the ACF group < 5, the most common co-occurring polyp is hyperplastic polyp, and for the ACF group> 10 – adenoma. In the group of people without a polyp, ACF was not found> 10, and is most likely to have normal ACF in the rectum. (*P* < 0.05)Relative frequency of all types of polyps is the smallest for ACF number < 5 and the highest for ACF > 10 (Fig. 3).

**Fig. 4:**
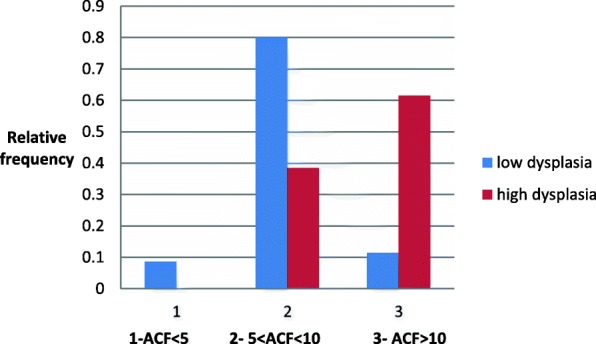
Probability occurrence of a type of dysplasia in the adenoma, depending on the number of ACFs in the rectum (ACF > 10)

The probability of developing small adenomas (up to 10 mm) is highest in the ACF < 5 group. The size of the co-existing adenoma in this group changes exponentially in the form of an equation *y* = 1, 1333*e*^−0, 693*x*^; with the indicator of determination *R*^2^ = 0, 999. When single ACFs are found in the rectum, we usually find small adenomas and no adenoma> 20 mm.In the 5 < ACF < 10 group, adenomas in the 10-20 mm range were most commonly found. The density distribution is normal in the form of an equation *y* = 0, 0305*e*^1, 0397*x*^ with a determination index *R*^2^ = 0, 9643. In ACF > 10 group, adenomas> 20 mm were most commonly found. The density distribution is exponential in the form of an equation y =  − 0, 3289x^2^ + 1, 1061*x* − 0, 3939 with a high indicator of determination *R*^2^ = 0, 999 (Fig. 4).

**Fig. 5:**
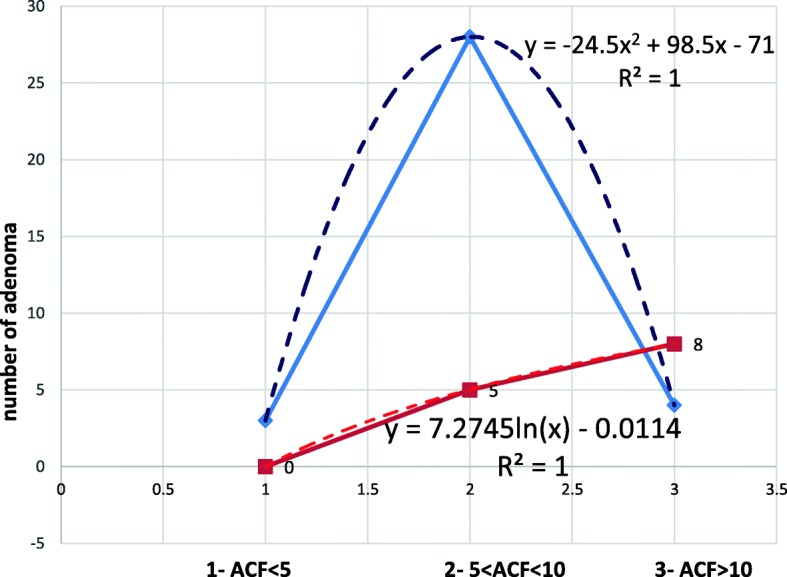
Distribution of the type of dysplasia in the adenoma depending on the number of ACF in the rectum. Red line- high dysplasia. Blue line- low dysplasia

People with high ACF (ACF > 10) are more likely to have adenoma with high dysplasia (statistically significant difference, *p* < 0.05). Adenomas with high dysplasia were not found in people with ACF < 5. In the group of people with 5 < ACF < 10 with adenoma, the most likely is low-grade dysplasia (statistically significant difference with significance level *p* < 0,05) (Fig. 5).

The distribution of adenomas with low dysplasia can be described by the equation y = − 24,5 × ^2^ + 98,x-71 with the determination index R2 = 1 (normal distribution)The distribution for adenomas with high-grade dysplasia can be described by the equation y + 72745ln(x)-0,0114 (the curve is logarithmic) with the determination index R^2^ = 1.

### Occurrence of polyps and CRC depending on the type of ACF in the rectum (Fig. 6)

The probability of occurence of dysplastic ACF is highest in the case of concomitant adenomas and the lowest for serated polyp with a statistically significant difference *p* < 0,001 (Table 2, Fig. 7).

**Fig. 6:**
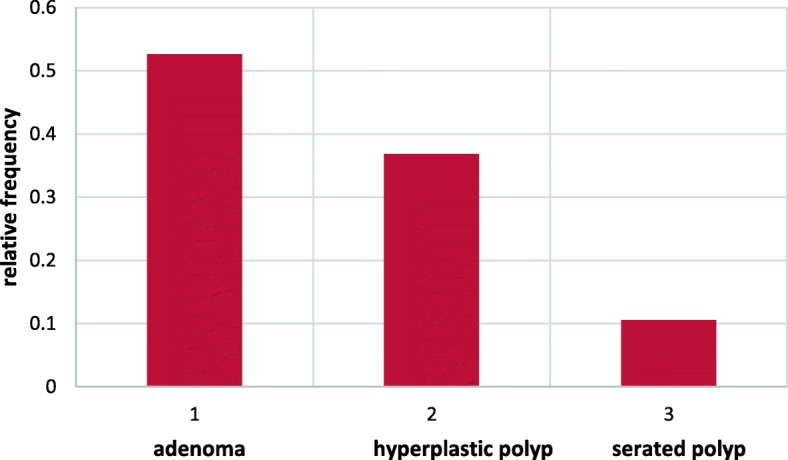
The probability of occurence of dysplastic ACF. Red- Dysplastic ACF

**Table 2:** Occurrence of polyps and CRC in various sections of the large intestine depending on the type of ACF in the rectum

	ACF normal				ACF hyperplastic				ACF dysplastic				ACF mixed			
Polyp location	R	SD	T	ACC	R	SD	T	ACC	R	SD	T	ACC	R	SD	T	ACC
Number	37	19	8	7	28	13	3	7	8	6	5	4	7	5	2	2
Type of polyp	Ad	Hy	Se	No	Ad	Hy	Se	No	Ad	Hy	Se	No	Ad	Hy	Se	No
Number	31	35	4	24	18	31	10	8	10	7	2	0	7	6	5	3
CRC location	R	SD		RC	R	SD		RC	R	SD		RC	R	SD		RC
Number	1	2		3	1	2		3	0	1		5	0	0		0

Polyp location: *R* rectum, *SD* sigmoid and descending colon, *T* transverse colon, *ACC* ascending colon and caecum. Type of polyp: *Ad* adenoma, *Hy* hyperplastic, *Se* serrated, *No* no polyp. CRC location: *R* rectum, *SD* sigmoid and descending colon, *RC* right colon

**Fig. 7:**
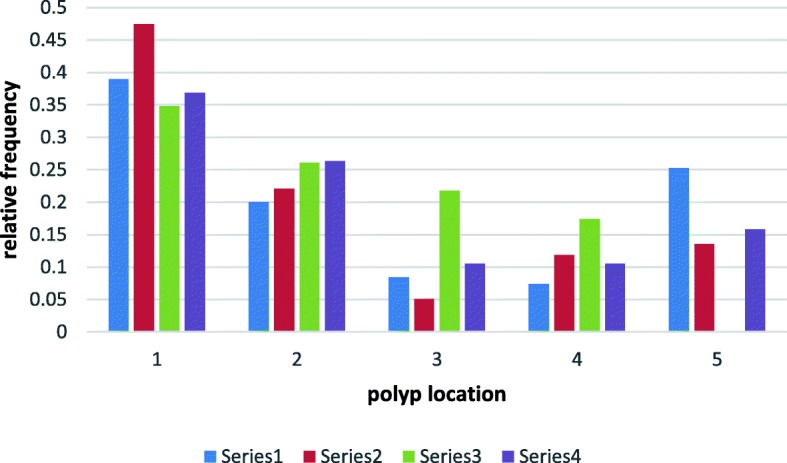
The probability of polyps distribution in all sections of the large intestine. 1-rectum 2- sigmoid colon, descending colon 3- transverse colon 4- ascending colon, caecum 5- no polypserie 1(blue)-normal ACF, serie 2(red)- hyperplastic ACF, serie 3(green)-dysplastic ACF, serie 4(violet)-mixed ACF

**Fig. 8:**
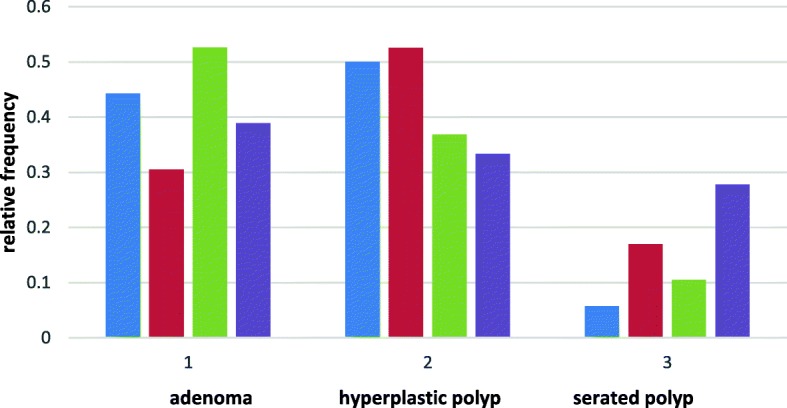
The probability distribution of different types of polyps depending on the type of rectal ACF. blue-ACF normal, red- ACF hyperplastic, green- ACF dysplastic, violet- ACF mixed

The probability of polyps distribution in all sections of the large intestine is asymmetrical. The maximum relative frequency (rf) for all types of polyps occurs in the rectum while in subsequent sections of the large intestine it decreases with varying intensity depending on the type of ACF in the rectum. The rf difference between polyps in the right half of the colon (location-3,4) for dysplastic ACD in the rectum compared to other types of ACF is statistically significant with a confidence level of *p* < 0,05. According to the chi^2^ criterion, the difference between the probability distribution of polyps in the large intestine for dysplastic and hyperplastic ACF is statistically significant with a significance level of *p* < 0,05. When no polyp was found in the large intestine, there was no dysplastic ACF and the difference in probability of other types of rectal ACF in this group of patients is not statistically significant (*p* > 0,05) (Fig. 8).

**Fig. 9:**
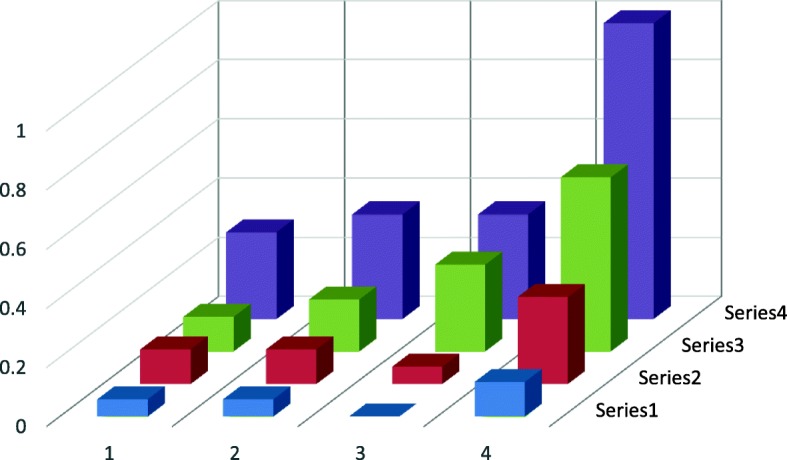
Relative distribution of the probability of CRC in different sections of the large intestine depending on the ACF rectal class. 1- ACF normal 2-ACF hyperplastic 3-ACF dysplastic 4- sum of events (1 + 2 + 3) Blue- rectum red- sigmoid colon + descending colon, green- transverse colon + ascending colon + caecum, violet- sum of events Series 1- conditional probability of rectal cancer for different types of ACF Series 2-conditional probability of CRC in sigmoid and descending colon for ACF types Series 3-conditional probability of CRC occurrence in the “right colon” for ACF types Series 4-conditional probability of CRC in different sections of the large intestine

Distribution of polyp types for ACF normal and ACF hyperplastic are similar to normal distribution. For ACF dysplastic and ACF mixed, the distribution of polyp incidence in the large intestine is similar to the exponential distribution.According to the chi^2^ criterion, the difference between these two types of probability distributions is statistically significant with a confidence level of *p* < 0,05.The probability of the type of polyp distribution in the large intestine depends on the type of ACF in the rectum.The most likely probability of colorectal adenoma is dysplastic ACF in the rectum.For adenomas, the difference between the probability of dysplastic and hyperplastic ACF in the rectum is statistically significant with a confidence level of *p* < 0.05. For other types, ACF is statistically insignificant with *p* > 0,05 (Fig. 9).

The comparison of the probability of conditional CRC formation in the “right colon” for dysplastic ACF is greatest in comparison to similar conditional probabilities of CRC formation for other types of ACF with a high significance level *p* < 0,05.

## Discussion

Colorectal cancer is a model example of a multi-stage carcinogenesis process, in which the advanced change occurs via the transformation of intermediate stages [4].

Colorectal adenomas are considered a precancerous condition for sporadic CRC forms, and their removal by use of endoscopic polypectomy correlates with a decrease in the incidence of CRC [9]. Currently, we are aiming at the detection of adenoma precursors and other polyps with possible potential for malignancy. ACF are considered precursors of colorectal adenomas. Multicentre studies have shown that there is a higher percentage of ACF in patients with CRC and adenomas as compared to patients with a regular large intestine [10]. Our study also shows that people with associated adenoma and CRC have a higher number of rectal ACF. The similarity of cellular kinetics and risk factors for ACF and colorectal cancer strengthens the hypothesis that they are pre-cancerous lesions.

Studies carried out by Figueiredo et al. show that apoptosis is significantly reduced in ACF in patients with a co-existing colorectal cancer who are over the age of 50 [11]. These results also show a substantial increase in the number of ACF from the age of 50. We presented similar data in another study [12]. As you can see in Fig. 2, the amount of ACF correlates well with the age of the respondents. Single ACF occur in the youngest individuals (ACF < 5). Since the age of 38 years, the number of ACF gradually increases to show a decreasing tendency since the age of 60 years. The number of 5 < ACF < 10 occurs slightly later, since the age of 50 years, and dynamically increases reaching the maximum at the age of 62 years, subsequently the increase is proportional. ACF > 10 occur at a more advanced age (55 years) and their number gradually increases with age (linear increase). The maximum number is observed at the age of 77 years.

The apoptotic index and cell kinetics are a measure for an occurrence of an abnormal cell division leading to the emergence of clones of pathological cells in a given focus. Over the age of 50, apoptosis in the ACF in patients with cancer is significantly lower, which may indicate a high risk of development of aberrant crypts in such patients.

In 1998, Takayama et al. [13] concluded that there is a significant relationship between the number of ACF, the presence of dysplastic ACF and the number of adenomas.

Dysplastic ACF constitute a direct precursor lesion of adenoma, because of their histological similarity, similar gene profile and clinical relationship with adenomas and CRC [13, 14].

The incidence of ACF in the same individuals is higher than that of adenomas and carcinomas.

We may, therefore, risk saying that in the majority of cases ACF constitute changes accompanying CRC and glandular polyps, and remain in full readiness to transform into a neoplastic lesion. The thesis of a significant oncological potential of ACF was demonstrated in the study by Siu et al. [15] which confirmed the presence of carcinoma in situ in ACF.

Various researchers report in their works a different incidence of dysplastic ACF. We find a smaller percentage of dysplastic ACF in the works of Nascimbeni, Adler, Rudolph [16–18], while values similar to our study group are demonstrated in Takayama’s work [6]. Moreover, there are studies where this percentage is greater than in our study group. Siu et al. [19] report the incidence of dysplastic ACF amounting to 73% in the Afro-American group and 44% among “white Americans”. Differences in the amount of dysplastic ACF in comparable groups may result from ethnic, dietary and environmental differences [6, 17, 18]. As we showed in our other work [12], a greater number of ACFs occur in people with obesity (especially with BMI > 35 kg / m2), a diet with excess red meat and a low-residual diet, which is also confirmed by other authors [13, 15].

Otori et al. [20] showed that hyperplastic ACF (as well as dysplastic ACF) may also transform into microadenomas. Siu et al. [19] present interesting data in stating that dysplasia does not usually affect entire dysplastic ACF and may be present in it focally. This may be evidence in favour of the theory of clonal expansion where initially a single cell in a focus forms a clone of potentially “malicious” cells, while the remaining cells in the focus demonstrate only minor abnormalities. Until the study conducted by Otori et al. [20], researchers confirmed the coexistence of different types of ACF within a single focus, however they did not show the progression of hyperplastic ACF towards dysplastic and micro- adenoma [8, 16, 21]. Similarly, hyperplastic polyps, which were considered to be completely benign, were being underestimated until the descriptions provided by such researchers as, for instance, Jass, Hamilton, Urbański and others, who illustrated the potential of malignant transformation of hyperplastic polyps [1–3].

According to Orłowska et al. [22] hyperplastic ACF play an identical role in the process of carcinogenesis as dysplastic ACF. However, the route of cancer formation with their presence is different. Most researchers dealing with ACF have no doubt that dysplastic ACF may constitute precancerous lesions. Shouldn’t we, therefore, while analysing the studies of Otori et al. [20] and the data provided by Shpitz et al. [23] take a closer look at the hyperplastic ACF and cease to underestimate their seemingly benign character?

Although ACF are more common in the sigmoid colon and rectum than in the right side of the colon, dysplasia is more commonly observed in the ACF originating from the caecum and the ascending colon. This may be evidence that the biological features of the ACF located on the left and right side of the colon are different, as may be the development of cancers located in different parts of the large intestine.

What is puzzling is the presence of right-sided malignant tumours with high ACF densities in the rectum [24]. If rectal ACF are not the precursors to neoplastic lesions located outside the rectum, only one explanation comes to mind. Namely, it is the effect of intestinal carcinogens that are active in the entire large intestine. Although they accumulate mainly in the final section of the large intestine and stimulate the formation of cancerous cells (the most common location of the ACF, polyps and malignant neoplasms of the large intestine), their activity also encompasses other sections of the intestine.

Little is known about the factors that initiate and stimulate the ACF growth in humans. It seems highly probable that if cancer emerges in the ACF-adenocarcinoma sequence, the same factors that cause cancer development should cause the development of ACF. With age, man acquires more and more new mutations. Statistically, in the elderly the number of detected ACF and malignant tumours increases. Works evaluating the ACF epidemiology list the same factors that contribute to the formation of the ACF, adenomas and colorectal cancer [6, 8, 16–18, 21].

A very significant fact is that in people over 80 years old the number of ACF no longer increases. On the contrary, there is an observable decrease in their number [12]. Presumably, in individuals over the age of 80 an atrophy of the mucous membrane occurs in the distal section of the large intestine, with the regeneration process not being as intense as in younger people. The fact that in this age group colorectal cancer is often diagnosed is rather the result of a slower growth of cancer that had not been recognised before in elderly patients.

Hurlstone et al. [25] report that the incidence of dysplastic ACF increases starting from patients with benign lesions through adenoma to CRC. It is believed that rectal dysplastic ACF can occur with carcinomas located both on the left and right side. A different opinion is expressed by Siu et al. [19] according to whom dysplastic ACF more commonly accompany CRC with right-sided location. According to Shpitz et al. [23] sporadic cancers are most frequently accompanied by normal and hyperplastic ACF, and the number of dysplastic ACF is higher in individuals with adenomas than in people with a regular large intestine. In their study Bouzorene et al. [26] show that colorectal cancer is most commonly accompanied by dysplastic ACF.

In the examined group, the presence of rectal ACF accompanied benign changes in the right half of the colon in 20.69% of cases, whereas CRC in 100%. If we consider malignant tumours in the study group, the right-sided location was observed in 50% of cases, while the right-sided location from the splenic flexure of the colon was noted in nearly 20% of the benign lesions found.

If we were to perform in the study group only rectoscopy or sigmoidoscopy it would be evidenced that more than 20% of patients have had mild lesions and 4.2% malignant lesions overlooked. The regular development of colonocytes is controlled by appropriate genes. The abnormalities in their expression and the impact of harmful environmental factors may be conducive to carcinogenesis, and the already initiated process may occur synchronously in various places in the large intestine. Stevens et al. [27] also found correlations between an occurrence of ACF with the positive history of advanced adenoma (adenomas larger than 10 mm, high grade dysplasia and villous component). A greater incidence of ACF in patients with a positive history of advanced adenoma is also reported by Rudolph et al. [18] and Takayama et al. [6]. In the Japanese study this relationship was statistically significant (*p* < 0.001). Takyama et al. [6] and Hurlstone et al. [25] demonstrated the evident dependence between the number of ACF and the number of polyps found in the same patients, whereas Figueiredo et al. [11] manifested that it also depends on the degree of dysplasia in the adenoma.

In the study group, along with an increase in dysplasia in ACF-associated adenoma, there was a greater number of ACF in the rectum. As in other studies [6, 25], also in our study group a higher number of ACF was found in people with a greater number of co-occurring polyps, which is particularly evident in people with three or more polyps. People who did not have a polyp in the large intestine had mainly less than 5 ACF. In the ACF > 10 group there is the highest probability of finding a large number of polyps in the large intestine compared to other groups (ACF < 5 < ACF < 10). The maximum relative frequency for this group (f / *n* = 0.5) and the exponential nature of the curve depicting this ACF group may indicate greater dynamics of the neoplasmatic process for this group of patients. This suggests that special attention be paid to such people and monitoring through control colonoscopy.

According to Roncucci et al., a higher ACF density is found not only in colorectal carcinomas and single adenomas, but also in patients with FAP [8]. In their study, Pretlow et al. show that a higher density of ACF and the occupying by them of larger areas of the large intestine as compared with sporadic cancers is observed in patients with Gardner syndrome (30). These data point to the fact that ACF occur in greater numbers and densities in patients with hereditary diseases of the large intestine which are associated with a greater risk of malignant transformation in these patients.

Taking into account the studies assessing the location of ACF in the large intestine and their density in particular sections of the large intestine, as well as the concurrent benign and malignant changes, it can be concluded that the determination of rectal ACF is a useful marker for the presence of neoplastic lesions in the proximal and distal sections of the large intestine [6, 17, 23–25].

The question may be asked whether the assessment of rectal ACF brings any specific benefits to the patient or relevant clinical information for the physician performing the examination. And if so, who should perform the evaluation of rectal ACF? Hurlstone et al. [25] report that the determination of rectal ACF is a useful prognostic factor in determining the likelihood of an occurrence of flat and recessed neoplastic lesions in the right half of the colon. Seike et al. [24] in his work shows that the number of ACF significantly predicts the presence of synchronous tumours of the large intestine localised proximally and distally.

In the study group, the number of rectal ACF’s is a good predictive factor for determining the likelihood of polyps in the large intestine. The presence of dysplastic ACF in the rectum increases the likelihood of developing right-sided polyps from the splenic flexion of the colon.

Analyzing the data from our study, it can be seen that if the rectum is found to be ACF normal and we do not find ACF dysplastic, in such people we usually do not find polyps in the large intestine.

Similar to the work of Seike et al. [24], we have shown that the probability of CRC in the right half of the colon for dysplastic ACF is highest compared to other types of ACF with a high level of significance *p* < 0.05.

Therefore, is it necessary to perform a “full” colonoscopy in all patients with a large amount of rectal ACF? Everything indicates that it is.

Patients with dysplastic ACF in the rectum should undergo a full colonoscopy as they commonly accompany malignant lesions and adenomas located proximally from the splenic flexure of the colon.

Dysplastic ACF are considered to be actual precursors of colorectal cancer, whereas other ACF as concurrent, such that they are also likely to progress to dysplastic ACF [28]. Based on literature analysis, Stevens et al. concluded that rectal ACF are a good prognostic factor for an occurrence of colorectal cancer in the future [27].

In view of the above data, the ACF should be looked at more closely and their seemingly mild character should not be disregarded, given the saying of the doctor and poet William Carlos Williams, “that which is possible is inevitable.”

## Conclusions

Rectal ACF are a useful marker for the presence of cancerous lesions in the proximal and distal sections of the large intestine.

## Data Availability

Data are in additional supporting file, for more materials please contact author for data requests.

## Abbreviations

ACF: Aberrant crypt foci
APC: Adenomatous polyposis coli
CRC: Colorectal cancer
FAP: Familial adenomatous polyposis
KRAS: KRAS Proto-Oncogene
p53: phosphoprotein p53,p53 tumor suppressor protein
